# Effects of Antenatal Glucocorticoid Therapy on Hippocampal Histology of Preterm Infants

**DOI:** 10.1371/journal.pone.0033369

**Published:** 2012-03-23

**Authors:** Deodata Tijsseling, Lia D. E. Wijnberger, Jan B. Derks, Cindy T. J. van Velthoven, Willem B. de Vries, Frank van Bel, Peter G. J. Nikkels, Gerard H. A. Visser

**Affiliations:** 1 Perinatal Center, University Medical Center, Utrecht, The Netherlands; 2 Department of Obstetrics and Gynaecology, Rijnstate Hospital, Arnhem, The Netherlands; 3 Laboratory for Neuroimmunology and Developmental Origins of Disease, University Medical Center, Utrecht, The Netherlands; 4 Department of Pathology, University Medical Center, Utrecht, The Netherlands; Erasmus University Rotterdam, Netherlands

## Abstract

**Objective:**

To investigate if antenatal glucocorticoid treatment has an effect on hippocampal histology of the human preterm newborn.

**Patients and Methods:**

Included were consecutive neonates with a gestational age between 24 and 32 weeks, who were born between 1991 to 2009, who had died within 4 days after delivery and underwent brain autopsy. Excluded were neonates with congenital malformations and neonates treated postnatally with glucocorticoids.

The brains were routinely fixed, samples of the hippocampus were stained with haematoxylin and eosin and sections were examined for presence or absence of large and small neurons in regions of the hippocampus. Additional staining with GFAP, neurofilament and vimentin was performed to evaluate gliosis and myelination. The proliferation marker Ki67 was used to evaluate neuronal proliferation. Staining with acid fuchsin-thionin was performed to evaluate ischemic damage.

**Results:**

The hippocampi of ten neonates who had been treated with antenatal glucocorticoids showed a lower density of large neurons (p = 0.01) and neurons irrespective of size (p = 0.02) as compared to eleven neonates who had not been treated with glucocorticoids. No difference was found in density of small neurons, in myelination, gliosis, proliferation or ischemic damage.

**Conclusion:**

We found a significantly lower density of neurons in the hippocampus of neonates after antenatal glucocorticoid treatment. Although the pathophysiological and clinical interpretations of these findings are not clear, they are consistent with those from experiments in mice and rhesus monkeys.

## Introduction

Administration of glucocorticoids (GCs) to pregnant women at risk of preterm birth has led to a major improvement in the outcome of preterm born neonates. Treatment with antenatal GCs reduces the risk of neonatal death, respiratory distress syndrome, periventricular/intraventricular haemorrhage, necrotising enterocolitis, infectious morbidity, need for respiratory support and neonatal intensive care unit admission [1]. Betamethasone and dexamethasone, the synthetic GCs that are given for this indication, cross the placenta and have a high affinity for the GC receptors. These receptors are found in most organs including the brain [2]. GCs promote cellular differentiation at the expense of proliferation resulting into dose related effects in many different species [3]. Regarding the brain there is a dose related reduction in weight in for instance fetal sheep and rat pups [4]–[6]. In humans, a reduction of head circumference after multiple antenatal GC courses has been found in various observational studies [7]–[9] and in one out of four recent large randomised controlled trials in which head circumference was an outcome measurement [10]–[13]. The effects of GCs on brain cell proliferation are most pronounced in areas undergoing active growth and differentiation at the moment of treatment [14]. In this respect, the hippocampal structure, which is part of the limbic system, plays a crucial role in cognitive functions such as learning, memory storage, and spatial orientation and is a metabolic active structure [15], [16]. Furthermore, receptors for GCs are highly expressed in the hippocampus [2]. It is therefore likely that among various brain regions the hippocampus appears to be the most vulnerable to antenatal GC treatment. Disturbance in development of the hippocampus may have effects later in life. In monkeys, multiple doses of dexamethasone were associated with a dose-dependent decrease in the number of neurons in the hippocampus as well as degeneration of neurons in this region [17], [18]. A study in mice using a clinically relevant dose has established a link between prenatal exposure to dexamethasone and reduced hippocampal volume as well as a reduced number of hippocampal neurons shortly after treatment [19]. Follow-up at adulthood of offspring mice who received corticosteroid treatment in utero showed decreased cell proliferation in the dentate gyrus and adverse effects on hippocampal function [20].

As far as we know, there are no data on the effects of antenatal GC therapy on hippocampal histology of the human fetus or neonate yet. It was our aim to investigate if antenatal GC treatment affects the histological structure of the hippocampus of the fetus and the neonate.

## Methods

### Ethics Statement

The study protocol was approved by The Ethics Committee of the University Medical Center, Utrecht. They concluded that the Medical Research Involving Human Subjects Act did not apply for this study and that the parents of the research subjects didn't need to be asked for consent. Anonymous use of redundant tissue for research purposes is part of the standard treatment agreement with patients in our hospital.

### Population

From a database containing data on all children born in the University Medical Center, Utrecht, The Netherlands, we identified consecutive neonates with gestational age between 24 and 32 weeks, born between 1991 to 2009, who died during or within 4 days after delivery and underwent a brain autopsy after oral informed consent of the parents. Neonates with congenital malformations or massive brain destruction, such as massive cerebral haemorrhage or encephalitis and/or treated with postnatal GCs were excluded.

We reviewed in retrospect the medical files of the included neonates and their mothers. We recorded the reason for admission, use of antenatal GCs, interval between the first gift of GCs and birth, use of postnatal GCs and other drugs, gestational age at delivery, route of delivery, birth weight, sex, parity of the mother, neonatal morbidity, underlying cause and mechanism of perinatal mortality and the interval between birth and death. A complete antenatal GC course consisted of two intramuscular doses of 12 mg betamethasone with a 24-hrs interval. Birth weight percentiles were calculated using birth weight z-scores. Growth charts corrected for gestational age, sex and parity according to the Dutch Perinatal Registry were used to calculate the birth weight z-scores (http://www.perinatreg.nl) [21]. Weight for GA at the 50^th^ percentile was used as the mean of the population and the average standard deviation (SD) calculated by the formula [−1SD+1SD]/2 was used. Subsequently, the z-score was converted into an exact percentile for each studied subject with a z-score to percentile web calculator (http://www.measuringusability.com). Underlying cause and mechanism of perinatal mortality was based on clinical and pathological findings classified using the Tulip classification [22].

### Histological preparation

Brain tissue was fixed in buffered formalin 4%. Four weeks after removal, coronal slices were made and embedded in paraffin after which 3 µm sections were cut and stained with haematoxylin and eosin (H&E). Subsequently the hippocampus was examined for the density of large, small and neurons irrespective of size in all four cornu-ammonal (CA) regions of the hippocampus (CA1, CA2, CA3 and CA4). The density of large and small neurons was scored using semiquantitative analysis comparable with the method used by Groenendaal et al. [23]: high density (4 points), moderate density (3 points), low density (2 points) or (nearly) absent (1 point). Density of neurons irrespective of size was scored by: high (4 points), moderate (3 points), low/moderate (2 points) or low (1 point).

In order to study possible effects of antenatal GC treatment on myelination and gliosis, we performed additional staining on 4 µm sections with antibodies reacting against neurofilament (Monosan, 1∶800), vimentin (Dako, 1∶400) and glial fibrillary acidic protein (GFAP, Dako, 1∶800) on the hippocampus of 11 of the 21 neonates (Table S1, numbers 2-5, 7-9,11,14,15,21). The sections were examined for intensity of staining in all four cornu-ammonal (CA) regions of the hippocampus (CA1, CA2, CA3 and CA4) and scored as: high (4), moderate (3), low (2) or (nearly) absent (1).

Proliferative activity of neurons was evaluated using the proliferation marker Ki67 (Dako, 1∶400). The Ki67 protein is expressed in all proliferating cells in late G1, S, G2 and M phases of the cell cycle [24]. Sections were examined for the number of Ki67 positive nuclei in the dentate gyrus and germinal matrix, two areas where active proliferation usually takes place. The number of Ki67 positive immunoreactive cells was scored as: high (4), moderate (3), low (2) or (nearly) absent (1).

To visualise neuronal degeneration and hypoxic ischemic damage a combined staining procedure, acid fuchsin-thionin (0.1% fuchsin, Gurr/BDH; 0.25% thionin, Chroma) was used. Neurons showing ischemic cell change with acidophilic (pink) cytoplasm and contracted nuclei were regarded as nonviable. Sections were examined for intensity of staining, markers of acute ischemia as apoptotic figures and cytotoxic edema in the hippocampus and scored for every category as: high (4), moderate (3), low (2) or (nearly) absent (1).

In order to test the hypothesis that small neurons are mature neurons and large neurons immature neurons, we performed an additional staining with antibodies reacting against doublecortin (1∶200, Santa Cruz). Doublecortin is a microtubule-associated protein expressed by neuronal precursor cells and immature neurons.

An experienced perinatal pathologist who was blinded for the treatment of GCs and for the other clinical data, performed the histological examination.

We studied the relation between hippocampal histology and the use of antenatal GCs. Furthermore, relations between the density of large, small or neurons irrespective and gestational age at delivery (≤28 weeks *versus* >28 weeks), birth weight (≤1000 grams *versus* >1000 grams), obstetrical complications (small for gestational age (weight<p10), preeclampsia (PE) and/or HELLP *versus* other pathology), mode of delivery (vaginal delivery *versus* caesarean section (CS)), placenta pathology (small placenta (weight <p10) with infarcts *versus* other pathology), signs of inflammation in the placenta (placenta with chorioamnionitis and/or funisitis *versus* no signs of infection in the placenta) and mechanism of death (respiratory insufficiency *versus* other pathology) were also investigated.

### Statistical analysis

Clinical characteristics between the groups were compared by the student t-test or Mann-Whitney U test. Histological data were analysed using the Mann-Whitney U test. SigmaStat software (SigmaStat for Windows, Version 2.0) was used for all statistical analyses. P values <0.05 were accepted as statistically significant.

## Results

A total of twenty-one neonates were included. Eleven neonates had not received GCs Ten had received a complete course of antenatal GC therapy. In Table S1, the clinical characteristics of the 21 included neonates are shown. Mean gestational age of the neonates not treated with GCs (n = 11) was 27.1 weeks (SD 2.4 weeks) and of the neonates treated with antenatal GCs (n = 10) 28.2 weeks (SD 2.1 weeks; p = 0.28). Median birth weight was 610 grams (range 450–1700 g) in the group not treated with GCs, 865 grams (range 475–1985 g) in the group treated antenatally.(p = 0.13). Median birth weight percentile was 10.1 (range 0.1–85) in the group not treated with GCs, 38.6 (range 0.4–80) in the antenatally treated group (p = 0.11). The median interval between the first dose of antenatal GCs and delivery was 3 days (range 1–16 days); one neonate had received a repeated course of GCs before birth (number 17).

**Figure 1:**
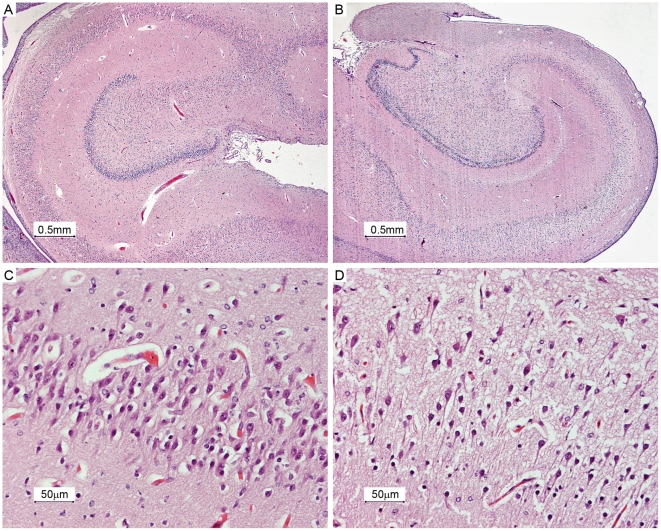
Example of the hippocampus of a patient not treated with GCs and of a patient treated with antenatal GCs. Figure 1A shows an example of the hippocampus of a patient not treated with GCs (number 6) and 1B of a patient treated with antenatal GCs (number 19). Panel 1C and 1D represent a magnification of transition zone 2 and zone 3 of panel 1A and 1B. Neuronal density is lower in Figure 1D compared to 1C. Figure 1A and 1B (20×, H&E) and Figure 1C and 1D (200×, H&E).

With routine H&E staining we examined in all neonates the presence or absence of large and small neurons in the different hippocampal regions (Figure 1A–D). The nuclei of large neurons showed hypochromatic staining and were large. When present, large neurons showed the highest density in the CA1 region and density gradually decreased in the CA2, CA3 and CA4 region. The nuclei of small neurons were small and round and showed a hyperchromatic staining. When present, small neurons had the lowest density in CA1 and the density increased gradually in the CA2, CA3 and CA4 region. We found no relation between the density of large, small or neurons irrespective of size and gestational age at delivery (≤28 weeks *versus* >28 weeks), birth weight (≤1000 grams *versus* >1000 grams), obstetrical complications (small for gestational age, PE and/or HELLP *versus* other pathology), mode of delivery (vaginal delivery *versus* CS), placenta pathology (small placenta with infarcts *versus* other pathology), signs of inflammation in the placenta (placenta with chorioamnionitis and/or funisitis *versus* no signs of infection in the placenta) and mechanism of death (respiratory insufficiency *versus* other pathology) (Table 1). In hippocampi of neonates treated with antenatal GCs, we found significantly more often a low density of large neurons (p = 0.01) as compared to hippocampi of neonates not treated with GCs (Table 1 and 2). No difference in small neurons was found (p = 0.35; Table 1 and 2). When we consider the density of neurons irrespective of their size, neonates treated with antenatal GCs showed a significant lower density of neurons in the hippocampus than untreated neonates (p = 0.02;Fig. 1C,D, Table 1 and 2).

**Table 1:** Density scores according to subgroups of patients characteristics.

	Score large neurons	p-value	Score small neurons	p-value	Score neurons irrespective of size	p-value
**Antenatal GC treatment**						
No AGCs	4.0 [3.0–4.0]	**0.01**	3.0 [1.3–3.0]	0.35	3.0 [3.0–3.8]	**0.02**
AGCs	2.0 [2.0–3.0]		2.0 [2.0–3.0]		2.5 [2.0–3.0]	
**GA at delivery**						
*</ = 28 weeks*	3.0 [2.0–4.0]	0.29	3.0 [2.0–3.0]	0.13	3.0 [3.0–3.5]	0.07
*>28 weeks*	3.0 [2.0–3.0]		2.0 [1.0–3.0]		3.0 [2.0–3.0]	
**Birth weight**						
*</ = 1000 grams*	3.0 [2.0–4.0]	0.19	3.0 [2.0–3.0]	0.27	3.0 [3.0–3.0]	0.10
*>1000 grams*	2.5 [2.0–3.0]		2.0 [1.0–3.0]		2.5 [2.0–3.0]	
**Obstetrical complications**						
*SGA, PE and/or HELLP*	3.0 [2.0–4.0]	0.68	3.0 [2.0–3.0]	0.18	3.0 [3.0–3.8]	0.09
*Other pathology*	3.0 [2.0–3.0]		2.0 [2.0–3.0]		3.0 [2.0–3.0]	
**Mode of delivery**						
*Vaginal delivery*	3.0 [2.0–4.0]	0.51	3.0 [2.0–3.0]	0.08	3.0 [2.8–3.0]	0.49
*Caesarean section*	2.5 [2.0–3.5]		1.5 [1.0–3.0]		3.0 [2.0–3.0]	
**Placenta pathology**						
*Small placenta (>p10) with infarcts*	3.0 [2.0–4.0]	0.78	3.0 [1.3–3.8]	0.56	3.0 [2.3–4.0]	0.30
*Other pathology*	3.0 [2.0–3.0]		2.0 [2.0–3.0]		3.0 [2.0–3.0]	
**Signs of inflammation in the placenta**						
*Chorioamnionitis and/or funisitis*	3.0 [3.0–3.3]	0.23	2.5 [2.0–3.0]	0.74	3.0 [3.0–3.0]	1.00
*No signs of inflammation*	2.0 [2.0–3.8]		2.0 [1.3–3.0]		3.0 [2.0–3.0]	
**Mechanism of death**						
*Respiratory insufficiency*	3.0 [2.0–4.0]	0.68	2.0 [2.0–3.3]	0.60	3.0 [2.8–3.3]	0.38
*Other pathology*	3.0 [2.0–3.5]		2.5 [1.5–3.0]		3.0 [2.0–3.0]	

Numbers represent the median scores, assigned for density of large, small and neurons irrespective of size (see text for details). Between square brackets: p25–p75 range.

Differences are tested with the Mann-Whitney U test.

**Table 2:** Density of large neurons, small neurons, and neurons irrespective of size in the hippocampus of 21 neonates.

	No GCs (n = 11)	Antenatal GCs (n = 10)	p-value
**Lage neurons**			
High	6	0	
Moderate	3	4	
Low	2	6	
(Nearly) absent	0	0	0.01
**Small neurons**			
High	2	0	
Moderate	5	3	
Low	1	6	
(Nearly) absent	3	1	0.35
**Neurons irrespective of size**			
High	3	0	
Moderate	7	5	
Low/moderate	1	4	
Low	0	1	0.02

Numbers represent individual cases.

Differences are tested with the Mann-Whitney U test.

The neurofilament and GFAP staining showed the same low intensity in all hippocampal regions. The intensity of the vimentin staining was the highest in the CA4 region in all hippocampi. In one neonate (number 4) the vimentin staining could not be evaluated because of absence of the hippocampal structure in the additional sections. Because we found no differences at all between the first eleven studied cases for the neurofilament, vimentin or GFAP staining, we terminated this investigation.

**Figure 2:**
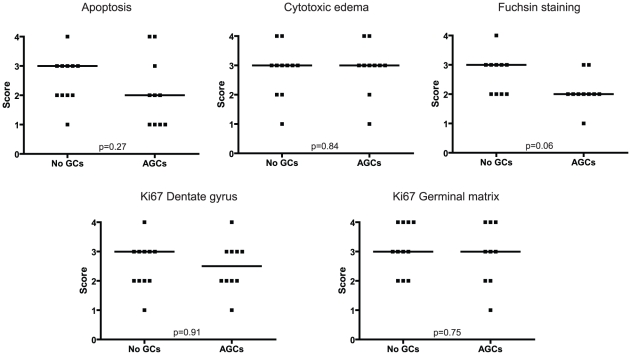
Apoptosis, cytotoxic edema, fuchsin staining and Ki67 positivity in the hippocampus and Ki67 positivity in the germinal matrix of preterm neonates not treated with antenatal GCs and treated with antenatal GCs. Horizontal axis: the two study groups, vertical axis: staining score (see text for details). Median values are indicated by horizontal lines. Differences are tested with the Mann- Whitney U test.

We found no differences in the degree of hypoxic ischemic damage by comparing the presence of apoptotic figures (p = 0.27), degree of cytotoxic edema (p = 0.84), intensity of fuchsin stained neurons (p = 0.06), number of Ki67 positive neurons in the dentate gyrus (p = 0.91) and germinal matrix (p = 0.75), between the group treated with antenatal GC and the group not treated with GC (Fig. 2, Table 3). In one neonate (number 20) the Ki67 staining could not be evaluated in the germinal matrix and in one neonate (number 1) the fuchsin staining could not be evaluated in the hippocampus, both because of absence of these structures in the additional sections.

**Table 3:** Semiquantitative analysis of the degree of apoptosis, cytotoxic edema fuchsin staining and Ki67 positivity in the hippocampus and of Ki67 positivity in the germinal matrix.

	No GCs (n = 11)	Antenatal GCs (n = 10)	p-value
Apoptosis	3.0 [2.0–3.0]	2.0 [1.0–3.0]	0.27
Cytotoxic edema	3.0 [2.3–3.0]	3.0 [3.0–3.0]	0.84
Fuchsin staining	3.0 [2.0–3.0]^1^	2.0 [2.0–2.0]	0.06
Ki67 staining dentate gyrus	3.0 [2.0–3.0]	2.5 [2.0–3.0]	0.91
Ki67 staining germinal matrix	3.0 [2.3–4.0]	3.0 [2.0–4.0]^2^	0.75

Numbers represent the median scores, assigned for high/moderate/low or (nearly) absent and the p25–p75 range between square brackets. Differences are tested with the Mann-Whitney U test.

1 n = 10 cases.

2 n = 9 cases.

Small and large neurons were both doublecortin immunoreactive. In the brains with most severe damage using acid thionin-fuchsin staining, we detected less doublecortin immunoreactive neurons, supporting the loss of neurons due to hypoxic ischemic damage.

## Discussion

To our knowledge, this is the first study evaluating the effects of antenatal GC therapy on the histology of the *human* hippocampus. We found a significantly lower density of neurons in the hippocampus of neonates after antenatal GC treatment (p = 0.02). No effect was found on gliosis or myelination after antenatal GC therapy in the eleven cases in which this was studied.

A limitation of this study is the heterogenicity of our patient group. Several pathological conditions during pregnancy, delivery and early neonatal life may have caused hypoxic ischemic damage in the fetus or neonate, a condition in which hippocampal neuronal damage can easily occur. These potentially confounding effects can only be ruled out in a large cohort study with a controlled prospective design. However, for obvious reasons it will not be possible to realise a study with such a design in human neonates. In our study we tried to minimise confounding factors in several ways. First, by including only neonates, who lived for a maximum of four days, limiting influences of early neonatal life. Furthermore, we compared groups for indicators of recent damage, by performing a staining with acid fuchsin-thionin, which highlights neurons with recent hypoxic ischemic damage. No significant differences were found between the two groups. The mode of delivery may affect the density of neurons, but there was no difference in neuronal density between the group born by a vaginal delivery compared to the group delivered by caesarean section.

The results of our study are consistent with earlier investigations using animal models [17]–[19]. In mice, antenatal dexamethasone treatment significantly decreased the number of neurons in the CA regions of the hippocampus directly after treatment until postnatal day 10 and in the dentate gyrus even till postnatal day 20 [19]. Also in two of the three studies investigating the effect of antenatal GC treatment on the hippocampus of rhesus monkeys, a decreased number of pyramidal neurons was found, which was ascribed to antenatal GC treatment. Beside this, shrunken neurons with pycnotic nuclei were found in the treated group [17], [18]. In the third study these phenomena were found in the treated as well as in non-treated animals and the findings could therefore not be ascribed to the GC treatment [25].

The mechanism behind the decreased neuronal density was not unveiled in this study. Several animal studies have focused on GC-induced hippocampal damage, however very few of them have used clinically relevant doses. Two studies that did use a clinically relevant dose, mention two possible mechanisms; suppression of proliferation *and/or* caspase-3 mediated apoptosis. However these studies are inconclusive. In 2003 Scheepens *et al.* [26] published a study determining the effects of a single course of prenatal betamethasone (2 doses of 170 µgram/kg) within the rat. They demonstrated that betamethasone was antiproliferative to brain cells, including hippocampal cells shortly after treatment for up to 4 days, with some catch-up recovery of the anti-proliferative effects there-after. No changes in caspase-3 mediated apoptosis were seen. In contrast Noorlander *et al.* [19] used a single, somewhat higher, dose of antenatal dexamethasone (1 dose of 400 µgram/kg) to investigate the effects on hippocampal development in mice. They detected, shortly after treatment, beside a decreased proliferative activity in the subgranular zone of the dentate gyrus, the zone where neurogenesis predominantly occurs, increased caspase-3 apoptotic activity throughout the whole hippocampus. The precise mechanism behind the anti-proliferative effect of GCs on the brain are unknown but possibly involve a down regulation in expression of the trophic growth factors: brain-derived neurotrophic factor (BDNF), IGF-1 and basic fibroblast growth factor (bFGF) [27]–[29]. Furthermore, administration of any of these three factors has been shown to specifically increase neurogenesis [30], [31]. In our study we found no difference in proliferative and apoptotic activity between the antenatal treated group and the control group. An explanation could be that the time interval between corticosteroid exposure and brain analysis may have been too long to detect a possible increase in apoptosis. Another explanation could be that the examined groups were too small to detect a difference.

There was no evidence of a reduction in myelination in our study. However since there is very little myelination in the immature brain between 24 and 32 weeks of gestation, we can not speculate on the possible effects of GCs on myelination given the very low gestational age of the neonates studied. Quantification of myelination at two years of age would be a more appropriate time to examine the effects of antenatal GCs on myelination.

We observed large and small neuronal cells in the hippocampus and both cell types showed its own distribution pattern in the hippocampal regions (large neurons highest density in CA1; small neurons highest density in CA4). By light microscopic examination, both cell types appeared to be neurons of the pyramidal layer of the hippocampus consisting of large and small pyramidal neurons. Our data showed that perinatal GC treatment has more effect on the density of large neurons than on the density of small neurons. We hypothesize that the large neurons might be more immature neurons and the small neurons mature neurons. To test this hypothesis we conducted a doublecortin immunoreactive staining. Both, large and small neurons were doublecortin immunoreactive. Hence, in our study the pathophysiological significance of the density of large and small neurons was not unveiled. Therefore, we also provided the density of all neurons, irrespective of size, which was also lower in the infants treated with GCs (Table 2).

The functional consequences of the effects on hippocampal neuronal density that we observed are unknown. In mice, decreased cell proliferation after exposure to GCs in utero has functional consequences like failure in a number of cognitive and behavioral functions in later life and accelerated aging [20]. However, caution is necessary when extrapolating data from animal models to the human situation.

A study that compared brainweight of neonates who had been delivered between 24 and 34 weeks of gestation showed no significant difference in brain weight between the group that received single or multiple courses of antenatal GCs and a group that did not receive GCs [32]. Thus far, long term outcome after one course of antenatal GCs has been reassuring, and shows only subtle neurological impairment at the age of 6 [33], but normal development in terms of physical, intellectual and emotional development at the age of 12, 20 and 30 years [34]–[37]. Repeated courses of antenatal GCs, however, have been associated with psychomotor delay and hyperactivity in childhood. [38]. Moreover, GC therapy after birth, with higher dosages and prolonged administration as compared to the antenatal therapy, has been shown to increase neuro-developmental impairment [39].

In conclusion, in this unique dataset we found a lower density of neurons in the hippocampus of preterm neonates who were antenatally treated with GCs. We cannot rule out the fact that the observation is caused by other factors as well. Determination of a causal relationship would require a randomized trial. Long-term effects are hard to predict because effects may be transient and the plasticity of the brain at this age may perhaps compensate for a decrease in neurons in the hippocampus. Our observations may cause concern because of the wide use of GC treatment in the perinatal period and indicate that more detailed and specific follow-up studies in humans are required.

## Supporting Information

Table S1 **Clinical characteristics of 21 included neonates.** Abbreviations:AGCs = antenatal glucocorticoids, Admin. = administration; CS = caesarean section; GA = gestational age at delivery; SGA = small for gestational age (weight <p10); PE = preeclampsia; PPROM = preterm premature rupture of membranes; TTTS = twin to twin transfusion syndrome; NA = Not available. ^*^ Birth weight in grams. ^1^ Enrolled in HELLP trial (high dose prednisolone versus placebo). ^2^ Repeated courses antenatal GCs.
(DOC)
