# Supplementary material for: Effects of Antenatal Glucocorticoid Therapy on Hippocampal Histology of Preterm Infants
Source: PLoS One. 2012 Mar 23;7(3):e33369. doi: 10.1371/journal.pone.0033369 (PMC3311632; doi:10.1371/journal.pone.0033369)
Supplement: Table S1 — Clinical characteristics of 21 included neonates. Abbreviations:AGCs = antenatal glucocorticoids, Admin. = administration; CS = caesarean section; GA = gestational age at delivery; SGA = small for gestational age (weight <p10); PE = preeclampsia; PPROM = preterm premature rupture of membranes; TTTS = twin to twin transfusion syndrome; NA = Not available. * Birth weight in grams. 1 Enrolled in HELLP trial (high dose prednisolone versus placebo). 2 Repeated courses antenatal GCs. (DOC) [file pone.0033369.s001.doc]

**Table S1. Clinical characteristics of 21 included neonates.**

|  | AGCs | GA | Birth weight^*^(Z-score/percentile) | Interval first admin. AGCs-birth (days) | | Interval birth-death (days) | Obstetrical pathology | Underlying cause of death; subclassification; mechanism^22^ | Pathologic examination of the placenta |
| --- | --- | --- | --- | --- | --- | --- | --- | --- | --- |
| 1 | No | 24.3 | 520 (-1.78/p3.8) | - | 0 | | SGA, PPROM, vaginal delivery | Infection; ascending; respiratory insufficiency | Chorioamnionitis, funisitis umbilicalis |
| 2 | No | 24.6 | 450 (-2.99/p0.1) | - | 0 | | PE, SGA, Doppler abnormalities, vaginal delivery | Placenta; placental bed pathology; respiratory insufficiency | Small placenta (weight <p10), infarcts (5% of placenta) |
| 3 | No | 24.7 | 540 (-1.28/p10) | - | 0 | | PE, placental abruption, vaginal delivery | Placenta; placental bed pathology; respiratory insufficiency | Placental weight p10, placental abruption, infarcts (10% of placenta) |
| 4 | No | 24.7 | 780 (0.54/p70) | - | 2 | | Dichorionic twins, intra-uterine infection, vaginal delivery | Infection; ascending; cardiocirculatory insufficiency | Chorioamnionitis |
| 5 | No | 25.6 | 525 (-1.54/p6) | - | 0 | | HELLP syndrome, SGA, vaginal delivery | Placenta; placental bed pathology; respiratory insufficiency | Small placenta (weight <p10), infarcts (5% of placenta) |
| 6 | No^1^ | 26.9 | 665 (-0.72/p23) | - | 0 | | PE, HELLP syndrome,vaginal delivery | Placenta; placental bed pathology; respiratory insufficiency | Small placenta circumvallata (weight <p10), infarcts (<5% of placenta), increased maturation |
| 7 | No | 28.0 | 540 (-1.55/p6) | - | 4 | | SGA, Doppler abnormalities, CS | Placenta; placental bed pathology; respiratory insufficiency | Infarcts (10% of placenta), increased maturation |
| 8 | No | 28.6 | 610 (-1.40/p8) | - | 3 | | SGA, fetal distress, CS | Placenta; placental bed pathology; cardiocirculatory insufficiency | NA |
| 9 | No | 29.4 | 870 (-1.20/p11) | - | 2 | | Fetal distress, CS | Placenta; placental bed pathology; cardiocirculatory insufficiency | Signs of circulation abnormalities, increased maturation |
| 10 | No | 30.0 | 1700 (1.04/p85) | - | 2 | | Placental abruption, CS | Placenta; placental bed pathology; cerebral insufficiency | Signs of fetal thrombosis |
| 11 | No | 31 | 1210 (-0.56/p29) | - | 0 | | Polyhydramnios, monochorionic twins, vaginal delivery | Placenta; placental pathology-development; cardiocirculatory insufficiency | Monochorionic-diamniotic placenta with some connecting vessels |
| 12 | Yes | 25.6 | 475 (-2.65/p0.4) | 2 | 0 | | Dichorionic twins, SGA, vaginal delivery | Placenta; placental bed pathology; respiratory insufficiency | Diamniotic-dichorionic placenta, small placenta (weight <p10), infarcts (10% of placenta), increased maturation |
| 13 | Yes | 26.1 | 840 (-0.05/p48) | 2 | 1 | | Spontaneous preterm delivery, CS, first neonate of quadruplet | Prematurity/immaturity; preterm labour; respiratory insufficiency | Quadruplet placenta, no abnormalities |
| 14 | Yes | 26.4 | 550 (-1.33/p9) | 3 | 0 | | Monochorionic twins, SGA, spontaneous preterm vaginal delivery | Placenta; placental pathology-development; cardiocirculatory insufficiency | Monochorionic-diamniotic placenta, TTTS |
| 15 | Yes | 26.4 | 760 (-0.71/p24) | 3 | 0 | | PPROM, vaginal delivery | Prematurity/immaturity; PPROM; respiratory insufficiency | Chorioamnionitis |
| 16 | Yes | 27.7 | 890 (0.10/p54) | 4 | 2 | | PPROM, suspected intra-uterine infection, vaginal delivery | Prematurity/immaturity; PPROM; respiratory insufficiency | Chorioamnionitis, funisitis umbilicalis |
| 17 | Yes^1,2^ | 28.3 | 770 (-0.66/p26) | 16 | 1 | | Dichorionic twins, HELLP syndrome, vaginal delivery | Placenta; placental bed pathology; cardiocirculatory insufficiency | Diamniotic-dichorionic placenta, increased maturation, partial placental abruption |
| 18 | Yes | 29.1 | 1300 (0.24/p59) | 7 | 0 | | PPROM, vaginal delivery | Prematurity/immaturity; PPROM; cardiocirculatory insufficiency | Increased maturation, chorioamnionitis, funisitis umbilicalis |
| 19 | Yes | 30 | 1200 (-0.55/p29) | 4 | 3 | | Fetal distress, CS | Placenta; placental bed pathology; cerebral insufficiency; | Small placenta (weight <p10), infarcts (5% of placenta), partial placental abruption |
| 20 | Yes | 30.3 | 1565 (0.71/p76) | 4 | 0 | | PPROM, suspected intra-uterine infection, CS | Prematurity/immaturity; PPROM; cardiocirculatory insufficiency | Chorioamnionitis |
| 21 | Yes | 32 | 1985 (0.83/p80) | 3 | 4 | | HELLP syndrome, fetal distress, CS | Placenta; placental bed pathology; cardiocirculatory insufficiency | NA |
